# The use of big data in psychiatry – the role of pharmacy registries

**DOI:** 10.1192/j.eurpsy.2021.2096

**Published:** 2021-08-13

**Authors:** M. Gonçalves-Pinho, J.P. Ribeiro, A. Freitas, P. Mota

**Affiliations:** 1 Department Of Psychiatry And Mental Health, Centro Hospitalar do Tâmega e Sousa, Penafiel, Portugal, Penafiel, Portugal; 2 2d4h, Center for Health Technology and Services Research (CINTESIS), Porto, Portugal; 3 Departamento De Psiquiatria E Saúde Mental, Centro Hospitalar do Tâmega e Sousa, Guilhufe, Portugal

**Keywords:** Big Data, Psychiatry, Pharmacy, Database

## Abstract

**Introduction:**

Administrative databases (AD) are repositories of administrative and clinical data related to patient contact episodes with all sorts of health facilities (primary care, hospitals, pharmacies,…).The large number of patients/contact episodes with pharmaceutical facilities available, the systematic and broad register and the fact that AD provides Real-world data are some of the pros in using AD data.

**Objectives:**

To perform a narrative review on the role of Big Data pharmaceutical registries in Mental Health research.

**Methods:**

We conducted a narrative review using MEDLINE and Google Scholar databases in order to analyse current literature regarding the role of BigData pharmaceutical registries in Mental Health Research.

**Results:**

Administrative variables like drug names and prices may be used and linked to other clinical variables such as patients disease, in-hospital mortality, length of stay,(…). The use of electronic medical records may also contribute to systematic surveillance approaches like local or national pharmacovigilance strategies, identification of patients at risk of developing complications and software pop-up warnings related to medication dosage, duplication and lateral effects. The use of Big Data pharmaceutical registries allows to create predictive epidemiological models regarding drugs lateral effects or interactions and may help to perform pharmacovigilance phase 4 clinical trials. Its use may be applied to the optimization of clinical decision, monitoring of drug adverse events, drug cost and administrative monitoring and as surrogate measures of quality care indicators.

**Conclusions:**

Big Data use in pharmaceutical registries allows to collect large and important clinical and administrative data that may be later used in Mental Health care and research.

**Disclosure:**

No significant relationships.

